# Capsular polysaccharide inhibits adhesion of *Bifidobacterium longum* 105-A to enterocyte-like Caco-2 cells and phagocytosis by macrophages

**DOI:** 10.1186/s13099-017-0177-x

**Published:** 2017-05-01

**Authors:** Amin Tahoun, Hisayoshi Masutani, Hanem El-Sharkawy, Trudi Gillespie, Ryo P. Honda, Kazuo Kuwata, Mizuho Inagaki, Tomio Yabe, Izumi Nomura, Tohru Suzuki

**Affiliations:** 1 Faculty of Applied Biological Sciences, Gifu University, 1-1 Yanagido, Gifu, 501-1193 Japan; 2 Faculty of Veterinary Medicine, Kafr El-Sheikh University, Kafr El-Sheikh, 33516 Egypt; 3 Department of Chemistry and Biomolecular Science, Faculty of Engineering, Gifu University, 1-1 Yanagido, Gifu, 501-1193 Japan; 4 CALM_live Imaging Facility, Centre for Inflammation Research, University of Edinburgh, Edinburgh, 47 EH16 4TJ UK; 5 Department of Molecular Pathobiochemistry, Graduate School of Medicine, Gifu University, 1-1 Yanagido, Gifu, 501-1193 Japan; 6 United Graduate School of Drug Discovery and Medical Information Sciences, Gifu University, 1-1 Yanagido, Gifu, 501-1193 Japan; 7 Department of Gene and Development, Graduate School of Medicine, Gifu University, 1-1 Yanagido, Gifu, 501-1193 Japan; 8 Center for Highly Advanced Integration of Nano and Life Sciences, Gifu University (G-CHAIN), Gifu University, 1-1 Yanagido, Gifu, 501-1193 Japan; 9 United Graduate School of Agricultural Science, Gifu University, 1-1 Yanagido, Gifu, 501-1193 Japan

**Keywords:** *Bifidobacterium longum* 105-A, Capsular polysaccharides, Caco-2 cell line, RAW 264.7, Cell adhesion, Phagocytosis

## Abstract

**Background:**

*Bifidobacterium longum* 105-A produces markedly high amounts of capsular polysaccharides (CPS) and exopolysaccharides (EPS) that should play distinct roles in bacterial–host interactions. To identify the biological function of *B. longum* 105-A CPS/EPS, we carried out an informatics survey of the genome and identified the EPS-encoding genetic locus of *B. longum* 105-A that is responsible for the production of CPS/EPS. The role of CPS/EPS in the adaptation to gut tract environment and bacteria-gut cell interactions was investigated using the Δ*cpsD* mutant.

**Results:**

A putative *B. longum* 105-A CPS/EPS gene cluster was shown to consist of 24 putative genes encoding a priming glycosyltransferase (*cpsD*), 7 glycosyltransferases, 4 CPS/EPS synthesis machinery proteins, and 3 dTDP-L-rhamnose synthesis enzymes. These enzymes should form a complex system that is involved in the biogenesis of CPS and/or EPS. To confirm this, we constructed a knockout mutant (Δ*cpsD*) by a double cross-over homologous recombination. Compared to wild-type, the ∆*cpsD* mutant showed a similar growth rate. However, it showed quicker sedimentation and formation of cell clusters in liquid culture. EPS was secreted by the ∆*cpsD* mutant, but had altered monosaccharide composition and molecular weight. Comparison of the morphology of *B. longum* 105-A wild-type and ∆*cpsD* by negative staining in light and electron microscopy revealed that the formation of fimbriae is drastically enhanced in the ∆*cpsD* mutant while the *B. longum* 105-A wild-type was coated by a thick capsule. The fimbriae expression in the ∆*cpsD* was closely associated with the disappearance of the CPS layer. The wild-type showed low pH tolerance, adaptation, and bile salt tolerance, but the ∆*cpsD* mutant had lost this survivability in gastric and duodenal environments. The ∆*cpsD* mutant was extensively able to bind to the human colon carcinoma Caco-2 cell line and was phagocytosed by murine macrophage RAW 264.7, whereas the wild-type did not bind to epithelial cells and totally resisted internalization by macrophages.

**Conclusions:**

Our results suggest that CPS/EPS production and fimbriae formation are negatively correlated and play key roles in the survival, attachment, and colonization of *B. longum* 105-A in the gut.

**Electronic supplementary material:**

The online version of this article (doi:10.1186/s13099-017-0177-x) contains supplementary material, which is available to authorized users.

## Background

The intestinal tract is considered to be one of the most densely colonized ecosystems of the human body and is colonized by trillions of microorganisms shortly after birth [1–4]. These organisms have been shown to have a significant symbiotic role in human health and nutrition, preventing pathogen colonization and maintaining mucosal immunity while being provided with access to key nutrients and a stable growth environment in the human intestine [5]. To date, over 1000 microbial species, mostly bacterial and anaerobic, have been cultured from the human intestinal microbiome [6]. Of these, *Bifidobacteria* and *Lactobacilli* are two of the most dominant genera conferring specific health benefits on their host [7].

The term probiotic was defined by Roy Fuller in 1989 [8] as a live microbial feed supplement that positively affects the host animal by improving its intestinal microbial balance. Moreover, probiotic bacteria are reported to have immunomodulation effects [9], anti-allergic effects [10, 11], the ability to inhibit hyperglycemia [12], and anti-hypertensive activity [13]. *Bifidobacterium* is one of the most commonly used probiotics in dairy products for human consumption [14–16].

*Bifidobacteria* are members of the *Actinobacteria* phylum; they are gram-positive, non-motile, anaerobic, non-filamentous rods with ‘bifido’ (branching) shape [17]. It was first isolated from the feces of a healthy infant by Henry Tissier, a French pediatrician at the Pasteur Institute in 1899 [18]. In general, it colonizes the colon rather than small intestine and can be isolated from the intestinal tract and feces of mammals, birds, fishes, and insects [19–21]. The ingestion of *Bifidobacteria* effects the prevention of constipation [22], an increase in calcium absorption from the gut [23], a reduction in the relapse frequency of ulcerative colitis [24], inhibition of cancer cell growth [25], and tumor growth [26]. It also suppresses the inflammation by the production of serpin [27] and suppresses the growth of pathogenic bacteria, such as *E. coli* O157:H7, by the production of acetic acid [28].

Nevertheless, the molecular mechanisms by which *Bifidobacteria* maintain a niche within their host and provide these effects are mostly still unknown [29].

The production of surface exopolysaccharide by probiotic bacteria is one of the proposed mechanisms by which these beneficial microbes facilitate commensal–host interaction and infer reported health benefits [30, 31]. Interest in bacterial polysaccharides has stemmed from the use of these economically important carbohydrate polymers in the food and biotechnology sectors, as well as their implications for health [32, 33]. Most bacteria produce more than one extracellular surface polysaccharide, such as lipopolysaccharides (O-antigens), capsular polysaccharide (CPS), and exopolysaccharide (EPS). EPS is thought to be released from cell-wall CPS [34–40]; thus, distinguishing between CPS and EPS is difficult. CPS/EPS consists of branched repeating units of sugar or sugar derivatives; mainly d-glucose, d-galactose, l-rhamnose, d-mannose, *N*-acetylglucosamine, and *N*-acetyl galactosamine in variable ratios [41]. EPS can be classified into two groups, homo- or heteropolysaccharides, based on their monosaccharide composition [42]. In pathogenic bacteria, EPSs are thought to be critical in host–microbe interactions where they facilitate in adherence and colonization within the human host [43] and have a role in immunomodulation [44].

The precise biological role of EPS produced by commensal bacteria is less clear; however, recent work by Fanning et al. [31] reported a pivotal and beneficial role for EPS in modulating various aspects of *Bifidobacteria*–host interactions, including the ability of *Bifidobacteria* to remain immunologically silent and provide pathogen protection. Probiotic EPS is also reported to determine cell surface characteristics such as the formation of biofilms [45], the colonization [31], and immunomodulation [46]. It is also involved in the protection against toxic compounds [47], bacteriophages [48], osmotic stress, and strict conditions such as bile and acid [47]. It is reported that some *Bifidobacteria* produce the two (or more) types of EPS [49], and these molecules should have specific functions and play roles in their survival strategies.

The first publically available genome was that of *B*. *longum* NCC2705 [50], and the completed genomes of 29 *Bifidobacteria* are held within the Gene Bank database at the time of writing. However, there are limited reports in the literature with regard to the genetic analysis of EPS biosynthesis in *Bifidobacterium* species. The generation of gene-knockout mutants of *Bifidobacteria* has been hampered by their low transformation efficiencies [51, 52].

We have overcome this by ‘Plasmid Artificial Modification (PAM)’ by averting the restriction system of bacteria [53, 54] and a sophisticated shuttle vector, pKKT427 [53]. We also constructed a gene-knockout system using a temperature-sensitive plasmid [55], *pyrE*-dependent bidirectional selection marker [56], improved promoter [57] and oxygen tolerance [58]. By the combination of these molecular genetic tools, the construction of gene deletion in *Bifidobacteria*, using the double cross-over homologous recombination system, became available at the practical level [55, 56, 59].

*Bifidobacterium longum* 105-A, which belongs to *B. longum* subsp. *longum,* was isolated from healthy human feces, and showed exceptionally high transformation efficiencies [60]. The whole genome sequence has been determined in our recent report [61]. Together with the ability to produce gene-knockout mutants with strain *B. longum* 105-A, it has opened the possibility to study the genetic basis of EPS biosynthesis in *Bifidobacteria.*

The initial step of the EPS-unit synthesis is catalyzed by priming glycosyltransferase (*cpsD* gene) [62]. *B. longum* 105-A harbors both a *cpsD* gene and six genes encoding glycosyltransferases, significantly more than the number of glycosyltransferases reported for other *B. longum* strains. We show that the symbiotic and probiotic *B. longum* 105-A contains an EPS-encoding genetic locus responsible for the production of EPS that provides resistance to both bile and acid treatment and is involved in the interaction of *B. longum* 105-A with intestinal epithelial cells and has a role in the resistance of *Bifidobacteria* to macrophage phagocytosis.

## Results

### Identifying of EPS locus and annotation of the putative EPS biosynthetic cluster in *B. longum* 105-A

**Fig. 1:**
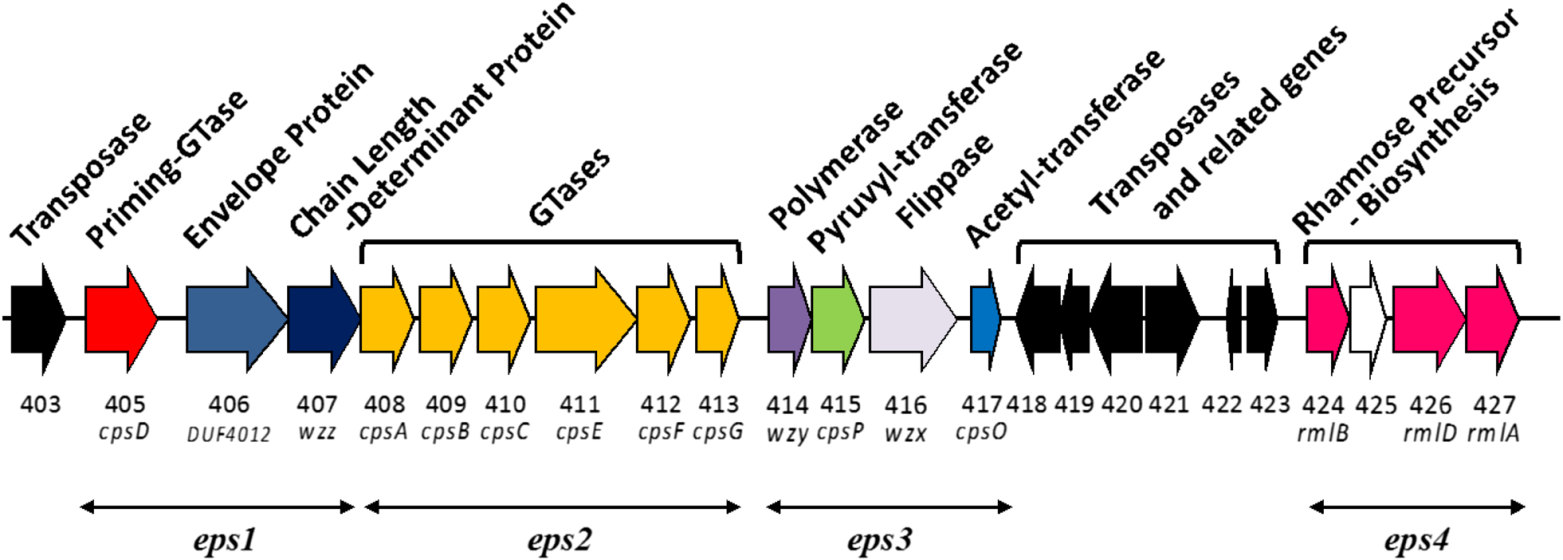
CPS/EPS gene cluster of *B. longum* 105-A. The gene cluster from *BL105A_0403* to *BL105A_0427*, which located nucleotide number 476,499–509,716, in genome sequence (AP014658) was shown. The enzyme and gene names, annotated by blast homology search (Table 1), were indicated. Putative operons, *eps1*-*4,* were shown with *arrows*

The genome of *B. longum* 105-A [61] harbors a putative EPS-encoding locus, which extends from *BL105A*-*0403* to *BL105A*-*0427* and encompasses a 33.2-kb region that harbors 24 genes predicted to be involved in EPS biosynthesis (Fig. 1; Table 1), four genes (*BL105A_0403, BL105A_0419, BL105A_0421*, and *BL105A_0422*) that encode a putative transposase, and three genes (*BL105A_0418, BL105A_0421*, and *BL105A_0423*) that encode a putative integrase. Half of these genes (i.e., 13 of 24) are organized into one gene set, while the remaining genes were organized into four smaller adjacent gene sets, putative operons, *eps1* to *4* (Fig. 1). The 1st gene set, encompassing *BL105A_0405* to *BL105A_0407*, is designated here as the *eps1* operon. The 2nd and largest set is *BL105A_0408* to *BL105A_0413,* as the *eps2* operon. The 3rd operon is *BL105A_0414* to *BL105A_0417*, as the *eps3* operon. The 4th set, *BL105A_0424* to *BL105A_0427,* is designated as the *eps4* operon. The EPS gene cluster of *B. longum* 105-A is modular in the organization, which is commonly reported for surface heteropolysaccharides [63].

**Table 1:** ORFs identified in the EPS gene cluster of *B. longum* 105-A

ORF	Gene	Size (bp)	Predicted encoded function	Best BLAST hit		Identity (%)
				Protein (accession no.)	Organism	
*BL105A_0403*		1272	*IS*30 family transposase	WP_008783747.1	MULTISPECIES	99
*BL105A_0405*	*cpsD welE*	1476	Undecaprenyl-phosphate galactose phosphotransferase; priming GTF	CCY95224	*B. longum* CAG:69	99
*BL105A_0406*	DUF4012	1730	Auxiliary protein for envelope protein	WP_008783638	*Bifidobacterium* sp.12 1 47BFAA	97
			Late cornified envelope protein 3C			
*BL105A_0407*	*wzz*	1460	Chain length determinant protein	WP_007057839.1	*B. longum*	97
*BL105A_0408*	*cpsA*	1154	GTF family 1	WP_023658085.1	*B. longum*	100
*BL105A_0409*	*cpsB*	1295	GTF	WP_041080238.1	*B. longum*	100
*BL105A_0410*	*cpsC*	1151	GTF	WP_008704446.1	*Ruminococcus* sp. 5 1 39 BFAA	68
*BL105A_0411*	*cpsE*	1964	Possible GTF	WP_009854068.1	*Streptococcus gallolyticus*	41
*BL105A_0412*	*cpsF*	890	GTF	WP_015131440.1	*Calothrix* sp.PCC 7507	29
*BL105A_0413*	*cpsG*	533	Possible GTF	WP_009854068.1	*Streptococcus gallolyticus*	42
*BL105A_0414*	*wzy*	797	Oligosaccharide repeat unit polymerase	WP_009854069	*Streptococcus gallolyticus*	34
*BL105A_0415*	*cpsP*	1100	Polysaccharide pyruvyl transferase	WP_004405434	*Vibrio nigripulchritudo*	26
*BL105A_0416*	*wzx*	1460	Flippase	CAI33492	*Streptococcus pneumonia*	41
*BL105A_0417*	*cpsO*	245	Maltose O-acetyltransferase domain protein	WP_009621938.1	*Desulfosporosinus* sp. OT	59
*BL105A_0418*	*Int*	834	Integrase catalytic region	BAP83067.1	*B. longum*	100
*BL105A_0419*	*istB*	834	Transposase (IstB-like ATP-binding protein)	BAP83067.1	*B. longum*	
*BL105A_0420*		1494	Integrase core domain	ALE36745.1	*B. longum*	100
*BL105A_0421*	*insE*	1549	Transposase (integrase)	WP_042764841.1	*Streptococcus pyogenes*	99
*BL105A_0422*	*insE*	408	Transposase	WP_047379062.1	*B. longum*	
*BL105A_0423*		669	Integrase catalytic region	WP_047379102.1	*B. longum*	97
*BL105A_0424*	*rmlB rfbB*	1022	dTDP-d-Glucose 4,6-dehydratase	WP_047379646.1	*B. longum*	98
*BL105A_0425*		1272	Hypothetical gene	WP_047379940.1	*B. longum*	99
*BL105A_0426*	*rmlC rfbC*	1457	Possible dTDP-4-keto-6-deoxy-d-glucose epimerase	WP_047379058.1	*B. longum*	94
*BL105A_0427*	*rmlA rfbA*	899	Glucose-1-phosphate thymidyl transferase	Glucose-1-phosphate thymidyly 1 transferase	*B. longum*	99

Located in the *eps1* operon, the *BL105A_0406* and *BL105A_0407* genes encode proteins that are annotated as an envelope protein and a chain length determinant protein, respectively, which have a high homology to the putative Wzd-Wze tyrosine kinase complex of *L. rhamnosus* GG [64]. The Wzb protein of *L. rhamnosus* GC has been biochemically characterized as a copper-dependent *O*-phosphatase [65]. In *Streptococci*, Wzd–Wze complex has been shown to be an autophosphorylating tyrosine kinase activity and has a regulatory role in CPS biosynthesis and polymer export [66].

This EPS cluster of *B. longum* 105-A contains 6 putative glycosyltransferases (GTF) genes encoding enzymes for the biosynthesis of EPS that are located in *eps2* operon and encompasses genes *BL105A_0408* to *BL105A_0413*. Comparable genome analysis of EPS clusters of some lactic acid bacteria species by Ruas-Madiedo et al. [62] revealed that the core of the EPS cluster is also occupied by GTF genes involved in the synthesis of repeating EPS units. The EPS oligosaccharide unit is built up by the sequential reaction of GTF that catalyze the transfer of the sugar moieties from activated donor molecules step-by-step. Although multiples of GTF genes have been reported in EPS clusters of other *B. longum* strains [17], *B. longum* 105-A is unique in having 6 GTF genes that range in size from 533 to 1964 bp. The first two putative GTF genes, *BL105A_0408* and *BL105A_0409*, have 100% amino acid homology to the previously identified *B. longum* GTF family 1 protein. The following four putative GTF genes, *BL105A_0410* to *BL105A_0413*, have amino acid homologies ranging from 68 to 29% with GTF genes of *Ruminococcus* sp., *Streptococcus gallolyticus*, and *Calothrix* sp. (Table 1) and probably encode the GTF of the remaining sugars of the EPS subunit in an ordered and glycosidic-linkage dependent fashion.

The putative *eps3* operon possesses *BL105A_0414* and *BL105A_0416* genes that encode for proteins that have homology to proteins involved in the polymerization and transport of repeated oligosaccharide units across the cytoplasmic membrane. The protein encoded by the *BL105A_0414* gene displays 34% identity with WP_009854069 from *S. gallolyticus* (Table 1), which is annotated as the oligosaccharide repeat unit polymerase Wzy. The *BL105A_0416* gene displays 41% identity with CAI33492 from *S. pneumonia*, which is interpreted as the flippase Wzx (Table 1). Polysaccharide subunits are flipped across the cytoplasmic membrane by the Wzx-type exporter and polymerized into long polysaccharides by Wzy-type polymerase at the outer-side cellular membrane (Fig. 1) [47]. The lower homology of the Wzx-type exporter and Wzy-type polymerase compared to the more conserved Wzd/Wze and Wzb homologs were also observed in the genome of *L. rhamnosus* GG [67]. Predicted results from SOSUI software show that the proteins of the genes *BL105A_0405* to *BL105A_0407* and *BL105A_0414* to *BL105A_0416* are all the transmembrane proteins (Additional file 1: Figure S2). However, the lower homology of putative Wzx and Wzy proteins is predicted to be due to these transmembrane proteins being specific for the associated EPS repeated unit of *B. longum* 105-A. ABC-transporter genes have also been reported to be involved in the transport of EPS oligosaccharide units across the cytoplasmic membrane in *Bifidobacterium* species [35, 58], but we did not find any genes in this *B. longum* 105-A putative EPS-encoding locus that had homology with known ABC-transporter genes.

Also, putative *eps3* operon contains *BL105A_0415* and *BL105A_0417*. The protein encoded by the *BL105A_0415* gene has 26% identity with WP_004405434 from *Vibrio nigripulchritudo* (Table 1), which annotated as a polysaccharide pyruvyltransferase. The protein encoded by the *BL1*05A_0417 gene has 59% identity with WP_009621938.1 from *Desulfosporosinus* sp. and was annotated as a maltose *O*-acetyltransferase. Pyruvyltransferases and acetyltransferase are thought to be involved in the modification of CPS/EPS, and should modulate physicochemical and biological properties.

Previous studies have shown that l-rhamnose is a component of Bifidobacterial cell walls [68]. In this study, we have identified three gene analogs known to be involved in the dTDP-rhamnose biosynthesis pathway located in the EPS gene cluster (Fig. 1). The genes *BL105A_0424, BL105A_0426* and *BL105A_0427* encode putative dTDP-d-glucose 4, 6-dehydratase, possible dTDP-4-keto-6-deoxy-d-glucose epimerase and dTDP-glucose pyrophosphorylase, respectively (Fig. 1; Table 1). Similarly, Lebeer et al. [64] also found putative genes for dTDP-rhamnose biosynthesis located in the EPS cluster of *L. rhamnosus* GG.

We observed that in the identified putative *B. longum* 105-A EPS gene cluster, the genes which encode the putative priming-glycosyltransferase, envelope protein, chain length determinant protein, first two glycosyltransferases, rhamnose biosynthesis precursors, and tyrosine kinase (*BL105A_0405*–*BL105A_0409*, *BL105A_0424*–*BL105A_0427*) are conserved in other *Bifidobacterium* strains, but other glycosyltransferases, polymerase, pyruvyltransferase, flippase, and acetyltransferase genes (*BL105A_0410*–*BL105A_0417*) are specific for the associated EPS repeating unit (36–37), as reflected by their lower level of similarity to other orthologs (Table 1), according to the reported mechanism of EPS production in *S. pneumoniae* and *L. rhamnosus* [64, 69].

### Morphology of *B. longum* 105-A Δ*cpsD* mutant

The priming-glycosyltransferase is predicted to be a necessary control point of CPS/EPS production. We hypothesized that the knockout of the gene *BL105A_0405*, a homolog of *cpsD*, could affect CPS/EPS production. To demonstrate this hypothesis, we knocked out the putative priming-transferase *cpsD* gene (*BL105A_0405*) in *B. longum* 105-A. The deletion of *cpsD* from *B. longum* 105-A was confirmed by PCR and DNA sequencing.

In contrast to the wild-type strain, which had a typically smooth and glossy colony appearance when grown on MRS plates, the mutant colony was less smooth and glossy. The growth curves of the wild-type strain and the *B. longum* 105-A ∆*cpsD* mutant strain were similar (Additional file 2: Figure S4A). However, the cells of the ∆*cpsD* mutant were found to quickly sediment after the stationary phase in liquid medium (Additional file 2: Figure S4B, C), while the wild-type strain remained in suspension (Additional file 2: Figure S4B, C).

To confirm EPS production of both the wild-type strain and the ∆*cpsD* mutant, we analyzed the supernatant from late exponential phase cultures by HPLC. The monosaccharides from the hydrolysate of wild-type *B. longum* 105-A EPS consisted of the monosaccharides that should be galacturonic acid, glucose (Glc), and galactose (Gal). Although two more monosaccharides were detected in the hydrolysate (Peaks 3 and 4 in Additional file 3: Figure S3A), we could not find out the details. According to Altmann et al. [70], the unusual sugar l-6-deoxyl-talose (l-6dTal) was found in the EPS produced by the *B. longum* subsp. *longum* 35624™ strain. Therefore, there is possibility that either Peak 3 or 4 in Additional file 3: Figure S3A, which was in the hydrolysate of the EPS from wild-type *B. longum* 105-A, was l-6dTal. The monosaccharides from the hydrolysate of the ∆*cpsD* mutant showed a marked decrease of Peak 3 and the disappearance of Peak 4 as compared to wild-type (Additional file 3: Figure S3A). On the other hand, the ratio of Glc/Gal was 1:1.48 in the EPS produced by the wild-type, while the ratio was 1:1.76 in the EPS produced by its ∆*cpsD* mutant (Additional file 3: Figure S3B). Furthermore, the EPS of the mutant contained polysaccharides with higher average molecular weight (500 kDa) than the wild-type (200 kDa) (Additional file 3: Figure S3C).

**Fig. 2:**
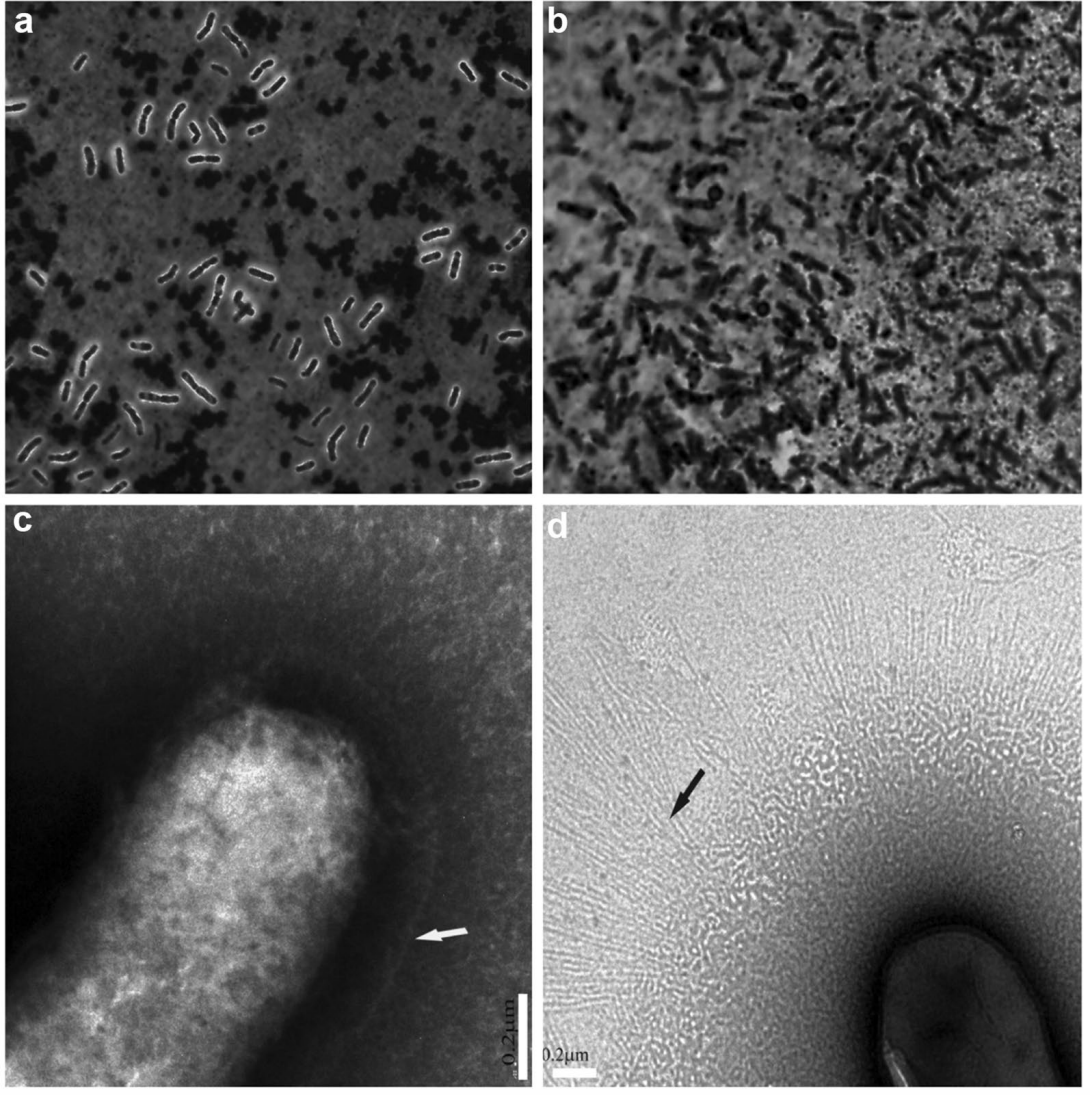
Light microscope images of *B. longum* 105-A and ∆*cpsD* mutant. India ink staining, the polysaccharide capsule appears as a clear halo around the microorganism in the wild-type *B. longum* 105-A (**a**), while this layer not present in its ∆*cpsD* mutant (**b**). TEM images with negative staining. Wild-type expressing ca. 0.2-µm-thick CPS layer (**c**, and *white arrow*), while the mutant did not possess the CPS but expressing long and dense fimbriae (**d**, and *black arrow*). *White bars* 0.2 µm

To determine the presence of an outer cell surface polysaccharide layer, presumed to be the capsule consisting of surface EPS, we performed India ink staining to visualize the polysaccharide layer. The polysaccharides in the capsule displace the colloidal carbon particles of the ink and appear as a clear halo around the microorganism [71]. It showed that a polysaccharide layer is absent in the ∆*cpsD* mutant bacterium, while a clear ‘polysaccharide’ halo could be observed in the wild-type bacterium (Fig. 2a). Our observation using a transmission electron microscope indicated that the wild-type *B. longum* 105-A is surrounded by a dense capsule layer (ca. 0.2 µm), while the *B. longum* 105-A ∆*cpsD* mutant has lost the CPS layer, however, an enormous number of fimbriae-like appendages were found instead of the capsule (Fig. 2b).

### qPCR analysis of EPS gene cluster

**Fig. 3:**
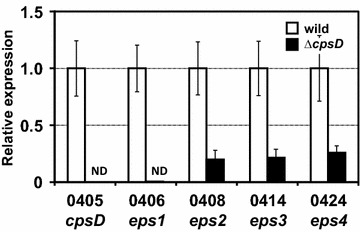
qPCR to confirm the deletion of *cpsD* and its influence on downstream genes. Relative expression of wild-type *B. longum* 105-A (*white*) against *B. longum* 105-A ∆*cpsD* (*black*) of *BL105A_0405* (*cpsD*), *BL105A_0406*, *BL105A_0408*, *BL105A_0414*, and *BL105A_0424* (as indicated *numbers*) were analyzed by qPCR using the 2 ^−∆∆CT^ method. The *rnaP* gene was used as housekeeping control to normalize the data. In comparison to the wild-type *B. longum* 105-A, the expression of *BL105A_0405* (*cpsD*) gene was undetectable in the Δ*cpsD* mutant. The downstream gene *BL105A_0406*, was significantly decreased by approximately 0.46% (*P* < 0.001) in the Δ*cpsD* mutant. The expression of genes, *BL105A_0408*, *BL105A_0414*, and *BL105A_0424*, were also decreased to 20% (*P* < 0.01). Temporal operon name also indicated below. *ND* not detected

In this point, the protein level verification, such as anti-CpsD antibody detection method, was unavailable; thus, an alternative approach was used to confirm the deletion of the *cpsD* gene and the influence of this deletion on downstream genes is shown in the qPCR analysis (Table 1). The expression of the *cpsD* gene was completely undetectable in the ∆*cpsD* mutant (Fig. 3). Thus, the deletion of the *cpsD* gene in the ∆*cpsD* mutant was also proven by qPCR. Expression of the late cornified envelope protein (*BL105A_0406*) gene, which is adjacent to the *cpsD* gene, was also undetectable in the ∆*cpsD* mutant. Interestingly, the expression of the *BL105A_0408* gene, encoding a GTF, and of the *BL105A_0414* gene (polymerase), which reside downstream of the *cpsD* gene in the *eps1* operon, was found to be significantly decreased, by approximately 80% (*P* < 0.001), in the ∆*cpsD* mutant. Furthermore, expression of gene *BL105A_0424* gene (dTDP-d-glucose 4,6-dehydratase) that resides in *eps4* operon was also decreased (*P* < 0.001) to about 20% (Fig. 3) in the ∆*cpsD* mutant compared to the wild-type strain expression.

### Tolerance to in vitro simulated gastrointestinal transit conditions

**Fig. 4:**
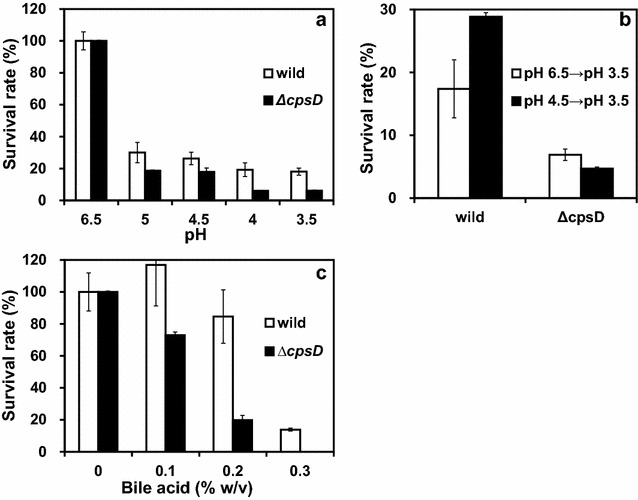
Effects CPS on the bacterial resistance to bile salts and acid. **a** Survival rate of *B. longum* 105-A (*white*) and *B. longum* 105-A ∆*cpsD* (*black*) in MRS broth, which is adjusted to indicated pH. **b** Adaptation by low pH. Cells were pre-incubated at 37 °C, for 2 h in MRS (pH 6.5; *white*) or pH 4.5 (*black*), then transferred to the same medium but pH 3.5 incubate for 2 h. *B. longum* 105-A (*left*) and *B. longum* 105-A ∆*cpsD* (*right*). **c** Survival rate of *B. longum* 105-A (*white*) and *B. longum* 105-A ∆*cpsD* (*black*) in MRS broth contained bile acid, 0–0.3% (w/v)

We compared the ability of the wild-type and the *B. longum* 105-A ∆*cpsD* mutant to survive in simulated gastrointestinal transit conditions. We found that a change from pH 6.5–5.0 significantly decreased the survival rate from 100 to 30% for the wild-type, and to 19% for ∆*cpsD* mutant strains (Fig. 4a). At a lower pH range, while the survival rate of the wild-type strain remained at approximately 20%, there was a significantly lower survival rate for the ∆*cpsD* mutant over a range of pH 4.0–3.5.

The pre-incubation treatment at pH 4.5 for 120 min significantly increased the survival rate of the wild-type strain challenged at pH 3.5 from 18 to 30% (Fig. 4b). Interestingly, with the ∆*cpsD* mutant strain that is lacking CPS, the pre-incubation acid treatment had no adaptation effect on the survival of rate of the bacteria challenged at pH 3.5.

We then investigated the protective role of *B. longum* 105-A CPS/EPS to exposure to bile salts. Incubation of wild-type *B. longum* 105-A in bile salt, up to a concentration of 0.2%, increased the overall colony survival rate, then it was decreased to 20% at a bile concentration of 0.3%, whereas the ∆*cpsD* mutant exhibited a significantly decreased survival rate with 0.1–0.3% bile salt (Fig. 4c).

### Binding to Caco-2 and internalization by macrophages

Cell surface CPS is an important bacterial adhesion factor, which assists inter-bacterial binding for biofilm formation, as well as adhesion to both inert and eukaryotic cellular surfaces [31, 72, 73]. In this study, we analyzed the role of CPS in *B. longum* 105-A binding on polarized intestinal epithelial-like cells Caco-2. To achieve this, we challenged the cells with a multiplicity of infections (MOI) = 100 from the *B. longum* 105-A wild-type and ∆*cpsD* mutant. The attached bacteria were determined after 1 h of incubation using Giemsa staining and a phase contrast microscope.

**Fig. 5:**
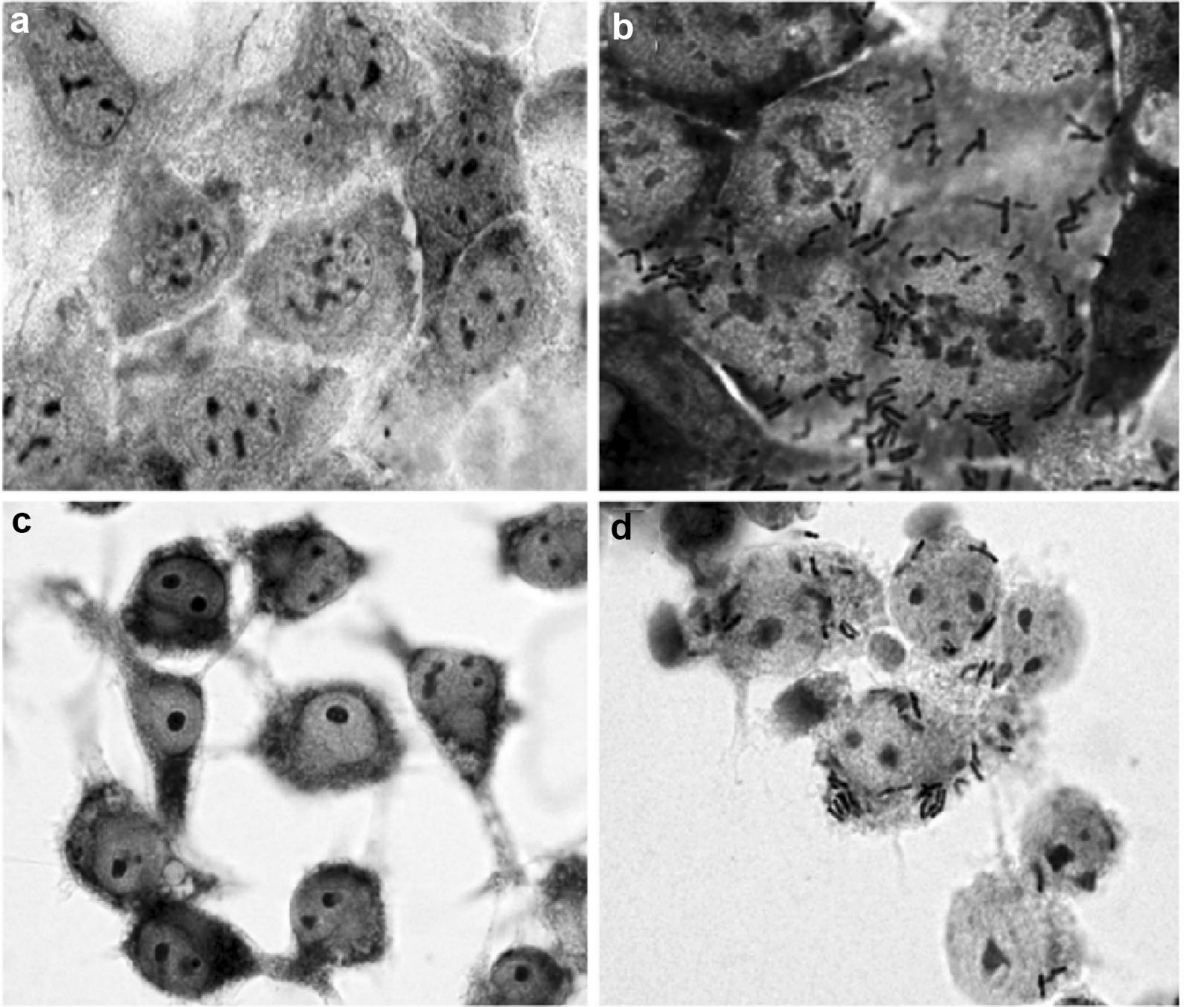
*Bifidobacterium longum* 105-A adherence to Caco-2 cells and phagocytosis by murine macrophage. 70% confluent monolayers of Caco-2 cells were challenged with *B. longum* 105-A (**a**) and its ∆*cpsD* mutant (**b**) at MOI = 100, then determined by phase contrast microscopy. No adherent bacterial cell was observed in the wild-type (**a**) but a lot of adherent bacteria were observed in ∆*cpsD* mutant (**b**). The number of attached ∆*cpsD* cells per Caco-2 cell was 7.8 ± 2.3 (nucleus ± SD). Three slides for each bacterial strain and at least 20 fields per slide were counted. *B. longum* 105-A phagocytosis by murine macrophage. Semi-confluent RAW 264.7 murine macrophage was challenged with *B. longum* 105-A (**c**) and its ∆*cpsD* mutant (**d**) for 30 min. Then, the medium was removed and the cells were washed 5 times with PBS and replace to DMEM containing gentamycin (100 µg/ml) and incubate for another 1 h. The coverslips were then washed 3 times with PBS and the cells were fixed with methanol and stained with Giemsa stain. In the wild-type, no bacterial cell was observed both inside and outside of macrophage cell (**c**) but ∆*cpsD* mutant was internalized into Raw 264.7 murine macrophage cells (**d**). The number of internalized bacterial ce1ls per macrophage cell was 4.1 ± 1

We found that the CPS was demonstrated to have a suppressive effect on the binding of the *B. longum* 105-A to the Caco-2 epithelial cell line. The wild-type bacteria did not bind to the monolayer cultured Caco-2 cells (Fig. 5a). However, its CPS mutant was able to bind (Fig. 5b), and the average number of adherent bacteria per nucleus, ±SD, was 7.8 ± 2.3 (Fig. 5b).

In addition, CPS is thought to play an essential role in interaction with non-specific immune cells. CPS has been implicated in the prevention of phagocytosis by phagocytic cells by *Lactococcus lactis* [74]. In the present study, optical microscopy using Giemsa stain was carried out to determine the role of CPS in interaction with murine macrophage cell line, RAW 364.7. The wild-type *B. longum* 105-A was shown to completely avoid the phagocytosis by murine macrophage (Fig. 5c). However, its *∆cpsD* mutant failed to evade internalization with this phagocytic cell, and a mean number of phagocytosed *B. longum* 105-A ∆*cpsD* per macrophage was 4.1 ± 1.4 (Fig. 5d).

## Discussion

The role of CPS in probiotic activities has been studied recently in *Lactobacillus and Lactococcus* probiotics [64, 74]. However, little is known about the function of the CPS/EPS in *Bifidobacteria*. In this study, we identified the EPS gene localization in *B. longum* 105-A and characterized the function of CPS/EPS in the attachment of the bacteria to intestinal epithelial cells and interaction with macrophage.

During the investigation of *B. longum* 105-A, we identified a gene cluster, which consisted of continuous 6 glycosyltransferases and one priming glycosyltransferase gene (Fig. 1). Among them, four genes are not found in other Bifidobacteria (Table 1). The putative genes involved in CPS/EPS biogenesis were also found in the flanking region including priming glycosyltransferase, *cpsD* (undecaprenyl-phosphate galactose phosphotransferase), oligosaccharide repeat unit polymerase, and flippase which was investigated in *L. rhamnosus* GG and found in some *Streptococci* [64]. The putative rhamnose biosynthesis genes *rmlA, rmlB, rmlD*, and putative pyruvyl transferase (*cpsP*) were also found in the downstream (Table 1). These findings suggest that *B. longum* 105-A produces CPS and/or EPS by this gene cluster, which is the likely polymer of a hepta-oligosaccharide unit. We assumed that this gene cluster was responsible for the CPS/EPS of this strain.

The bacteria use several strategies to adapt to their environment. One of the most important mechanisms is the cascade of long-term horizontal gene transfer between bacteria. To achieve this, the bacteria can acquire or lose genes through receiving plasmids, genomic islands, and bacteriophages. These methods can generate smaller insertion sequences (*IS)*, transposons and integrons that are associated with the integrases and transposases which are required for the generation of gene losses and the acquisition of new genes in the bacterial genome [75].

The gene cluster of *B. longum* 105-A is surrounded by multiple genes encoding putative transposase genes (*BL105A_0403, BL105A_0421* to BL105A_0422). Hidalgo-Cantabrana et al. [17] noted that the presence of genes encoding transposases and insertion sequences is a common feature found in the EPS clusters of both lactic acid bacteria species and *Bifidobacteria.* Moreover, this region shows low nucleotide homology to other *Bifidobacteria*. It suggests that this gene set has been gained from other bacteria by several horizontal gene transfer events in the recent evolution of *B. longum* 105-A. Interestingly, there are three putative integrase genes in this region. It is reported that the genome sequences of *B. longum* NCC2705 and DJO10A harbor the mobile integrase cassette (MIC) structures, consisting of three contiguous integrase genes flanked by an inverted repeat and a palindrome structure sandwiched by two *IS* elements. During a pure culture of *B. longum* DJO10A of over 1000 generations, a MIC element was found to be deleted from the genome along with ~50 kb of the sequence [51, 76, 77]. This EPS gene cluster of *B. longum* 105-A also harbors the three putative integrase genes and multiple transposase genes. It suggested that the active MIC structure exists in this EPS gene cluster, and deletion of this cluster may be caused during a pure culture of *B. longum* 105-A by prolonged culture.

The culture morphology of the bacteria on the plate indicated that the wild-type strain had a smooth and glossy colony appearance when grown on MRS plates, while the mutant colony was less shiny. Dertli et al. [47] reported that a single nucleotide change in the *epsC* gene of *L. johnsonii* FI9785 also caused a morphological change from a rough to a smooth colony.

The ∆*cpsD* mutant was quickly sediment after the stationary phase in the liquid medium due to loss of the capsule. The capsule removal allowed bacterial aggregation and facilitated the sedimentation in the liquid media (Additional file 2: Figure S4B, C), but the wild-type strain continued in suspension for a long period. Fanning et al. also observed that *B. breve* UCC2003 EPS negative mutants were found to sediment during growth in the liquid medium [31].

In this study, we found that although the *cpsD* was removed from *B. longum* 105-A, the EPS was still produced by the mutant, but some monosaccharides had a reduced amount. The data obtained from India ink staining and TEM postulated that these changes in the polysaccharide structure lead to changes in the morphology of the ∆*cpsD* mutant that are different from the wild-type (Fig. 2). Previous investigations have demonstrated that monosaccharide l-6-deoxy-talose (l-6dTal) plays an important role in capsule formation [78]. As there is much possibility that the monosaccharide, which had a reduced amount, is l-6dTal, this agreed with our results, and we suggested that the absence of the capsule in the *cspD* mutant was due to the decrease of some monosaccharides, such as l-6dTal, in its EPS secretion. It was documented that the appearance of adhesion factors, such as fimbriae and galactan, was correlated with changes in EPS structure and increased the cell–cell binding. The appearance of fimbriae-like appendages after removing the CPS is reported in *L. rhamnosus* GG [64], which agreed with our TEM analysis as the fimbriae-like structure was observed in the ∆*cpsD* mutant. Taken together, the data suggest that CPS inhibit fimbriae expression by the wild-type *B. longum* 105-A. Our qPCR finding indicated that the expression of the *BL105A_0406* gene was not detected in the mutant. Expression of the genes, which localized downstream of *the cpsD* gene, were found to be reduced. It suggested that the *cpsD* gene regulates the expression of other genes in the clusters [79].

After oral intake by the host, *Bifidobacteria* have to reach the colon, the suitable niche, and deal with the stresses throughout the gastrointestinal tract including the low-pH environment of the stomach and bile salts excreted in the duodenum [80, 81]. The pH of gastric acid is 1.5–3.5 in the human stomach lumen and gradually increases in the small intestine from pH 6 to about pH 7.4 in the terminal ileum. The pH drops to about 5.7 in the caecum but again gradually increases, reaching pH 6.7 in the rectum [82].

In general, the viability of *Bifidobacteria* at gastric juice pH values is known to be low [83], showing survival rates ranging from less than 1–30% depending on the experimental conditions, species, and strains. A mechanism for an adaptive response to low-pH exposure has been reported in lactic acid bacteria species [84]. However, there are only a few reports of acid tolerance induction of *Bifidobacteria* [83]. Our results suggest that pre-incubation of the wild-type *B. longum* strain at pH 4.5 significantly increased the survival rate of the wild-type strain challenged at pH 3.5 (Fig. 4b). These observations suggested that the pre-incubation treatment induced an adaptive pH response in the wild-type strain. On the other hand, pre-incubation acid treatment of the ∆*cpsD* mutant strain that is lacking CPS showed no significant effect on the survival of rate of these bacteria tested at a pH 3.5 (Fig. 4b), indicating that the mutant could not adapt to the low pH in vitro.

Bile salt is a detergent-like biological compound, which is stored in the gallbladder and flows to the duodenum that disrupts the structure of bacterial cell membranes. In the intestine, its concentration is retained in the range between 0.05 and 2% under the physiological conditions [85, 86]. The tolerance to bile salt is a necessary property for probiotic bacteria to survive transit through the duodenum [85]. Bile salt affects both the cell and colony morphology [87]; it shows that gut bacteria deal with it in several ways [87]. In addition to the active efflux and hydrolysis of bile salts, the changes in the architecture/composition of the capsular polysaccharide layer appear to be a prominent bile tolerance mechanisms in *Bifidobacteria* [85, 88, 89]. A correlation between *Bifidobacteria* EPS production and bile tolerance has been shown *to exist* in both in vitro *and* in vivo [31, 62]. The result of our resistance assays revealed that production of CPS by *B. longum* 105-A was shown to provide resistance to stress environmental conditions. Although the stress conditions in the colon are comparatively mild, we presume that EPS is needed to tolerate the strict stress conditions of the stomach and duodenum. This finding suggested that CPS/EPS exocellular polymer layers act as a protective coat against environmental conditions (Fig. 4c) [90, 91].

Attachment to epithelial cells by probiotic bacteria is a crucial event that allows for the colonization of the host intestinal tract. It allows the microbiota to multiply on the gut surface and perform its function of preventing of pathogenic bacteria from attaching. We used the human intestinal Caco-2 epithelial cell line [92, 93]. CPS is the major bacteria factor that has been implicated in the binding of many probiotics to the intestinal epithelium, and it was shown to play a role in *B. breve* colonization to mouse intestines [31]. We hypothesized that the presence of CPS inhibits expression of bacterial factors, such as fimbriae that might be involved in bacterial cell attachment. We verified this hypothesis by challenging the cultured Caco-2 cells with *B. longum* 105-A and its ∆*cpsD* mutant. This study demonstrates that the wild-type *B. longum* 105-A did not bind to the epithelial cells in comparison to extensive binding by its *cpsD* mutant (Fig. 5a, b). These results were completely different from that demonstrated by Fanning et al. 2012, which showed a significant role of CPS in the initial binding of *B. breve* in the initial colonization of the mouse gut. We explain that because of the CPS of *B. longum* 105-A is the completely has a different structure from that of *B. breve*. Previous studies have shown that deletion of the gene responsible for the production of long galactose-rich EPS in of *L. rhamnosus* GG resulted in an increased adhesion to Caco-2 cells [64, 94, 95]. The polysaccharides derived from *B. longum* 105-A showed unique and complete composition in comparison to other different probiotics bacteria. The CPS of *B. longum* 105-A might act as a barrier against colonization of this bacterial strain by covering or suppressing the expression of the organelles that help the bacteria to bind, such as fimbriae (Fig. 2c, d).

In Gram-positive pathogens, the CPS are considered to be the virulence factor that prevents phagocytosis. We performed phagocytosis experiments to assess the impact of *B. longum* 105-A’s CPS on preventing phagocytosis by macrophages. Our results showed that the *B. longum* 105-A CPS confers resistance to *B. longum* 105-A against being internalized by murine macrophages (Fig. 5c, d). The susceptibility to phagocytosis of CPS-negative mutant strains indicates that the CPS mutant can help in the activation of host immunity [96].

In summary, the results of this study identified the CPS/EPS gene cluster in *B. longum* 105-A. The CPS plays key roles in the survival of *B. longum* in vitro by conferring resistance to adverse physiological conditions such as low pH and bile salts. We also demonstrate that CPS production and fimbriae formation are negatively correlated. This work has confirmed that the CPS plays a crucial role in *B. longum* 105-A interaction with host cells. We speculate that l-6dTal is the predicted monosaccharide that plays a role in capsule formation. Future work should confirm the structure of *B. longum* 105-A CPS in relation to its function.

In this work, we have demonstrated that the ∆*cpsD* mutant of *B. longum* 105-A interacts better with the eukaryotic cells than the wild-type bacteria in vitro. In the future, further investigations, including in vivo study, should be carried out to examine its ability to survive in the animal host and to its role in immune modulation and preventing the infection with pathogenic microorganisms.

## Methods

### Bacterial strains and culture conditions

*Bifidobacterium longum* 105-A was used as the host strain. *Escherichia coli* TOP10 (Thermo Fisher Scientific, Waltham, MA, USA) was used as the cloning host for plasmid construction. *B. longum* was grown under anaerobic conditions at 37 °C in MRS broth (Becton, Dickinson and Company, Franklin Lakes, NJ, USA). *E. coli* were grown on LB medium (10 g tryptone, 5 g yeast extract and 5 g NaCl/L) at 37 °C. For plate culture, 1.5% agar was added to the medium before autoclaving. Chloramphenicol (25 µg/ml), spectinomycin (75 µg/ml), or ampicillin (100 µg/ml) was supplemented as necessary. The growth curve of *Bifidobacterium* was recorded using Bio-plotter OT-201 (Oriental Instruments, Sagamihara, Japan).

### *In silico* analysis

The genome sequence of *B. longum* 105-A was previously reported [61] (Accession Number: AP014658). Blast search [97] was performed using the National Centre for Biotechnology Information website (http://blast.ncbi.nlm.nih.gov/Blast.cgi). Sequence analysis was executed using CLC Genomics Workbench (Qiagen, Hilden, Germany) and motif searches were performed using the EMBL-EBI website (http://www.ebi.ac.uk/services). The hydrophobicity plot and transmembrane region predictions were carried out by SOSUI (http://harrier.nagahama-i-bio.ac.jp/sosui/) [98].

### Molecular techniques

Genomic DNA of *B. longum* 105-A and its derivatives were extracted by using an ISOIL for Beads Beating Kit (Nippon Gene Co., Ltd, Tokyo, Japan). Plasmid extractions from *E. coli* strains were performed using a QIAprep Spin Miniprep Kit (Qiagen). DNA digestion with restriction enzymes was performed according to the manufacturer’s protocol (Takara Bio Inc., Kusatsu, Japan). DNA sequencing of plasmid and genomic DNA was performed on an ABI 3130xl DNA sequencer using a BigDye Terminator ver. 3.1 Cycle Sequencing Kit (Thermo Fisher Scientific).

### PCR

The primer sequences used in this study for generating plasmids or confirming the deletion of target genes are described in Additional file 4: Table S1. The primers were designed by In Silico Molecular Cloning (IMC, In Silico Biology, Inc., Yokohama, Japan), plasmid Editor Software or Oligo Primer Analysis Software ver.7 (Molecular Biology Insights, Colorado Springs, CO, USA). GoTaq DNA polymerase (Promega, Fitchburg, WI, USA) was used for colony-direct PCR of *E. coli* transformants, and KOD-plus-Neo DNA polymerase (Toyobo Co. Ltd., Osaka, Japan) was used for cloning of *B. longum* genomic DNA, as described by the manufacturer’s protocol. The PCR products were analyzed by 1% (w/v) agarose gel electrophoresis with TAE buffer. For cloning, the target fragments of the PCR products were extracted from the gel using a NucleoSpin kit (MACHEREY–NAGEL, Düren, Germany). The PCR conditions using KOD-plus-Neo DNA polymerase were as follows: 94 °C for 2 min, 25 cycles of 98 °C for 10 s, 57 °C or 60 °C for 30 s, and 68 °C for 1 min. The PCR conditions using GoTaq DNA polymerase were as follows: 95 °C for 2 min, 30 cycles of (95 °C for 1 min, 57 °C or 60 °C for 1 min, and 72 °C for 1 min), then 72 °C for 10 min.

### Plasmid construction

The plasmid for the gene deletion, pKO403-∆*cpsD* (Additional file 5: Figure S1), was constructed as follows by using the Golden Gate Technique [99]. The PCR primers were designed according to the putative *cpsD* gene (*BL105A_0405*) of *B. longum* 105-A genome sequence. To obtain the gene deletion DNA fragments, about 1.0 kb up- and downstream fragments of *BL105A_0405* were amplified by PCR as summarized in Additional file 4: Table S1. To construct the gene deletion plasmid, we used the modified pUC19 and pKO403 *Bifidobacterium*-*E. coli* shuttle vector plasmids [55]. Both plasmids carry *lacZ* to facilitate blue-white selection (pUC19-*Sap*I and pKO403-*lacZ*). The pUC-*Sap*I plasmid was digested using *Bsp*68I, and the produced DNA fragments were connected to the *Bsp*68I-digested pUC-*Sap*I by ligation conducted at 16 °C for 30 min. The ligated plasmids were introduced into *E. coli* TOP10 and confirmed by colony PCR to obtain the correct constructs. These up- and down-stream plasmids and pUC-Sp were cloned into a pKO403-*lacZ* using *Sap*I and then transferred into *E. coli* TOP10 and confirmed by colony-direct PCR. Obtained plasmid was designated as the pKO403-∆*cpsD* plasmid.

### Gene knockout of target gene

*Bifidobacterium longum* 105-A cells were transformed with pKO403-∆*cpsD* by electroporation [60]. After electroporation, cells were spread and cultured on MRS plates containing Sp. The transformants were selected and cultured in MRS liquid medium containing Sp. The bacterial culture was serially diluted and spread on MRS plates containing Sp and was incubated at 42 °C for 3 days. The obtained colonies were duplicated onto Sp- and Cm-MRS plates and incubated at 37 °C for 2 days. Sp-resistant (Sp^r^) and Cm-sensitive (Cm^s^) colonies were selected as gene-knockout candidates and analyzed by PCR and DNA sequencing (Additional file 5: Figure S1).

### EPS characterization

The supernatant from late exponential phase cultures of *B. longum* 105-A wild-type or its ∆*cpsD* mutant was filtrated using a 0.45-µm filter. The polysaccharide of culture supernatant was precipitated using 2 volume of 99.5% ethanol for an overnight at 4 °C. The ethanol-suspended polysaccharides were centrifuged at 7000 rpm for 30 min. The pellet was dried out at 55 °C for 1 h and re-suspended in water. The obtained polysaccharides were then acid hydrolyzed with 4 M trifluoroacetic acid at 100 °C for 3 h. The chromatography of monosaccharide analysis was conducted with an Asahipak GS-220 HQ column (300 × 7.5 mm, Showa Denko, Tokyo, Japan). The EPS was eluted in water at a flow rate of 0.4 ml/min at 30 °C, followed by post-column reaction with arginine/boric acid, and monitored by a fluorescent detector (Excitation: 331 nm, Emission: 383 nm). The chromatography of monosaccharide analysis to separate the glucose and galactose was performed with a YMC-Pack NH2 column (250 × 4.6 mm, YMC Co., LTD., Tokyo, Japan). The EPS was eluted with 25% water and 75% CH_3_CN, at a flow rate of 0.5 ml/min at 50 °C with a fluorescent detection as above. For molecular weight analysis, the SUGAR KS-804 column (300 × 8.0 mm, Showa Denko, Tokyo, Japan) was used with a flow rate of 1.0 ml/min using a refractive index detector at 50 °C. The experiments were performed in duplicate.

### India ink staining

Twenty microliter**s** of the bacterial culture was mixed with one drop of India ink on microscope slide glass, left to dry, then stained with crystal violet for 1 min, then rinsed gently with water, and dried [71]. The slides were examined using bright-field illumination on a microscope (BX-51, Olympus Corp., Japan).

### Transmission electron microscopy (TEM)

For further identification of the morphology of *B. longum* 105-A wild-type and *B. longum* 105-A ∆*cpsD* mutant, we used the negative stain technique of TEM with phosphotungstic acid (PTA). Briefly, the bacteria were cultured overnight in MRS broth medium. Glow-discharged grids with a supported membrane (Cat. No. U1011, EM Japan Co. Ltd., Tokyo, Japan) were used to be covered by bacterial cells. 5 µl of the bacterial culture was placed onto the grid and incubated for 60 s, and then stained with 5 µl of 2% PTA (pH 7.4) for 45 s. The excess stain was drained using filter paper, and the grids were dried in a desiccator for 3 h. The bacteria were observed with a TEM at 200 kV (JEM 2100F, JEOL Ltd., Tokyo, Japan).

### Analysis of gene expression levels by qPCR

For qPCR analysis, total RNA was extracted from 10 ml logarithmic growth phase cultures (OD_660_ = 0.5–0.6) of *B. longum* 105-A wild-type and *B. longum* 105-A ∆*cpsD* mutant. The RNA was stabilized before extraction with RNA Protect Bacteria Reagent (Qiagen). The cells were enzymatically lysed with 2 mg/ml Labiase (Cosmobio, Japan) followed by mechanical disruption of the cells using a beads beader [100]. Total RNA was extracted using the RNeasy Mini Kit (Qiagen). The genomic DNA contamination was removed by DNase treatment for two times using RNase-free DNaseI (Takara Bio Inc.). Reverse transcription was performed using Script Reverse Transcriptase (Bio-Rad Lab. Inc., Hercules, CA, USA). Expression of the *cpsD* gene (*BL105A_0405*), *BL105A_0406* gene (encoding the putative envelope protein), *BL105A_0408* gene (encoding the putative glycosyltransferase), *BL105A_0414* gene (encoding the putative polymerase), *BL105A_0424* gene (encoding the putative dTDP-d-glucose 4,6-dehydratase) in both wild-type and mutant was quantified by qPCR on a real-time PCR system (StepOnePlus™, Thermo Fisher Scientific). The primers were designed using a primer analysis software (Oligo ver. 7, Molecular Biology Insights, Colorado Springs, CO, USA) to give amplicons of 80–150 bp (Additional file 4: Table S1). Calibration curves were prepared in triplicate for each pair of primers. Dissociation curves were used to check the specificity of the amplicons. The qPCR reaction was performed using the THUNDERBIRD^®^ SYBR qPCR Mix (Toyobo Co., Ltd.). The qPCR conditions were 95 °C for 20 s and 40 cycles of 95 °C for 10 s and 60 °C for 30 s. All samples were assessed in triplicate. The gene expression was compared to the housekeeping gene, *BL105A_1946* gene encoding RNase P, using the 2^−∆∆CT^ method [47].

### Resistance to low pH

To assess the resistance to Low pH, logarithmic growth phase culture (OD_660_ = 0.6) was harvested by centrifugation and re-suspended in fresh MRS medium, which had been adjusted to pH 3.5–5.0 with 6 N HCl. Cell suspensions were incubated at 37 °C for 2 h under anaerobic conditions. Then, they were serially diluted (×10^8^–10^10^) and plated on MRS agar plates. The CFU were enumerated after overnight incubation at 37 °C [101]. For pre-incubation treatments, cultures were incubated in a fresh MRS medium, which had adjusted to pH 4.5, at 37 °C for 2 h. The culture suspensions were then re-suspended in fresh MRS medium, adjusted to pH 3.5, and incubated at 37 °C for 2 h. The CFUs of the sample were enumerated as above.

### Resistance to bile salt

To assess the resistance to bile acid, the logarithmic growth phase culture (OD_660_ = 0.6) was harvested by centrifugation and re-suspended in a fresh MRS medium adjusted from 1.0 to 3.0 g/L with bile salt (Ox, Wako Pure Chemical Industries, Ltd., Osaka, Japan). Suspensions were incubated at 37 °C for 2 h and then serially diluted and plated on MRS agar plates. Plates were incubated overnight at 37 °C, and the CFU were enumerated. The experiments were performed in duplicate.

### Caco-2 cell line adhesion assay

To determine the role of *B. longum* 105-A EPS in binding to the Caco-2 cell line, we performed the experiments according to the method published by Guglielmetti et al. [102]. Briefly, Caco-2 cells were cultured for 2 weeks at 37 °C in 5% CO_2_ and moisture. This allowed for cellular differentiation and the development of microvilli. Caco-2 cells were then seeded onto 20 mm ϕ coverslip, which had been coated with mouse collagen IV (Trevigen, Gaithersburg, MA, USA), in 12-well plate at 10^5^ cells per well, 24 h before being challenged with the bacteria. The cells were washed twice with PBS and kept in DMEM without antibiotics for 1 h before bacteria were added. Bacteria were inoculated overnight on MRS broth. MRS broth cultures were centrifuged; the bacterial pellets were washed twice with PBS and then re-suspended into pre-warmed DMEM without antibiotics at an OD_660_ = 0.7. One milliliter of bacterial suspension was added to each well of Caco-2 cultures at a dose of 100 MOI, and then incubated anaerobically at 37 °C in 5% CO_2_ and moisture for 1 h. The cells were then washed 5 times with sterile PBS, fixed using 99.8% methanol, and washed twice with PBS. Then the coverslips were incubated for 20 min with Giemsa stain (Muto Pure Chemicals, Tokyo, Japan) at a 1:10 dilution. After several washes with PBS, the coverslips were mounted with mount-quick (Daido Sangyo Co., Ltd., Tokyo, Japan) and examined using phase contrast on a microscope (BX-43, Olympus Co., Tokyo, Japan).

### Macrophage phagocytosis assays

To determine the role of *B. longum* 105-A’s CPS in the interaction of the bacteria with non-specific immune cells, we performed the antibiotic protection assay as described previously by Chapot-Chartier et al. [74], with some modification. Briefly, we cultured RAW 264.7 murine macrophage cells in Dulbecco’s Modified Eagle Medium with high glucose (DMEM, Sigma-Aldrich, St. Louis, MO, USA) at 37 °C in a 5% CO_2_ and moisture. RAW 264.7 cells were seeded into collagen-coated coverslips in 24-well plates at 2.6 × 10^5^ cell/cm^2^. The cells were challenged with an MOI of 100 for 30 min. The non-internalized bacteria were removed by washing the cells five times with warmed PBS, and then 1 ml of DMEM medium containing 250 µg/ml gentamicin was added. The cells were incubated at 37 °C for 2 h to kill the extracellular bacteria. The cells were then washed and stained as specified above in Caco-2 cell line adhesion assays.

The Caco-2 and macrophage media were supplemented with 10% (v/v) heat-inactivated fetal bovine serum, 1.5 mM l-glutamine, 1/100 volume of non-essential amino acid (Sigma-Aldrich), 1/100 volume of Penicillin–Streptomycin (10,000 U/ml, Thermo Fisher Scientific).

### Statistical analysis

The difference in the expression levels of the downstream genes of *cpsD BL105A_0406, BL105A_0408 BL105A_0414*, and *BL105A_0424* was analyzed by the Student t test.

## Additional files

**Additional file 1: Figure S2.** SOSUI prediction of the membrane protein in *B. longum* 105-A. The protein of the gene *BL105A_0405* (*cpsD*) to *BL105A_0407* and *BL105A_0414* to *BL105A_0415* are the membrane protein.

**Additional file 2: Figure S4.** Characteristic features of *B. longum* wild-type strain and its ∆*cpsD* mutant in growth medium. (a) The growth curves of the wild-type *B. longum* strain and the *B. longum* 105-A ∆*cpsD* mutant strain were similar. (b) The cells of ∆*cpsD* mutant quickly sediment after stationery phase in liquid medium while the wild-type strain remained in suspension. (c) Measurements OD 660nm of wild-type *B. longum* strain and the *B. longum* 105-A ∆*cpsD* mutant over a panel time point grown ii liquid culture without agitation; the detected decrease in OD values for *B. longum* 105-A ∆*cpsD* mutant is due to cell sedimentation.

**Additional file 3: Figure S3.** HPLC analysis of EPS derived from *B. longum* 105-A wild-type its Δ*cpsD* mutant. A: Chromatogram of the monosaccharides in hydrolysates of the EPS from wild-type *B. longum* 105-A (blue line) and *B. longum* 105-A Δ*cpsD* mutant (red line). The chromatography was performed with Asahipak GS-220 HQ column (300 × 7.5 mm) and monitored by a fluorescent detector (Excitation: 331 nm, Emission: 383 nm). Peak 1, galacturonic acid; Peak 2, glucose and galactose; Peak 3 and 4, are unknown. B: Chromatogram of the monosaccharides in hydrolysates of the EPS from wild-type *B. longum* 105-A (blue line) and *B. longum* 105-A *ΔcpsD* mutant (red line) for separating the glucose and galactose. The chromatography was performed with YMC-Pack NH2 column (250 × 4.6 mm) and monitored by a fluorescent detector (Excitation: 331 nm, Emission: 383 nm). The ratio of Glc : Gal was 1 : 1.48 in the wild-type, while it was 1 : 1.76 in its *∆cpsD* mutant. Peak 1, glucose; Peak 2, galactose. C: Chromatogram of the molecular weight distribution of the EPS from wild-type *B. longum* 105-A (black line) and *B. longum* 105-A *ΔcpsD* mutant (red line). The chromatography was performed with SUGAR KS-804 column (300 × 8.0 mm) and monitored by a refractive index detector. Average molecular weight: Peak 1, 500 kDa; Peak 2, 200 kDa.

**Additional file 4: Table S1.** Primers used in this study.

**Additional file 5: Figure S1.** Schematic presentation of gene Knockout construction of *cpsD*. About 1 kb length upstream (*BL105A_403,* transposase) and downstream (*BL105A_406,* 406) regions were amplified and introduced into the franking regions of Sp^r^ marker on pKO403-Cm (1) (Sakaguchi), which carries temperature sensitive reprocation origin (Ori Ts) and Cm^r^ marker. The obtained plasmid (pKO403-∆*cpsD*) was introduced into *B. longum* 105-A, then selected on MRS+Sp plate at 42 °C. Obtained recombinants should consist of Sp^r^ and Cm^r^ single cross over (SCO) clones and Sp^r^ and Cm^s^ double cross over (DCO) clones. DCO clone was selected by by the replica selection with MRS+Sp and Cm plate. Obtained DCO clone was confirmed by PCR and DNA sequencing and designated *B. longum* 105-A ∆*cpsD.*

## Abbreviations

CPS: capsular polysaccharide
EPS: exopolysaccharide
bp: base pairs
CFU: colony-forming unit
OTU: operational taxonomic unit
PCR: polymerase chain reaction
PBS: phosphate buffered saline
DMEM: Dulbecco’s Modified Eagle Medium with high glucose
qPCR: quantitative PCR
Sp: spectinomycin
Cm: chloramphenicol
TEM: transmission electron microscopy
PTA: phosphotungstic acid
OD660: optical density at 660 nm
MOI: multiplicity of infection
MIC: mobile integrase cassette
HPLC: high-performance liquid chromatography
GTF: glycosyltransferase
