# Supplementary figures and images for: Capsular polysaccharide inhibits adhesion of Bifidobacterium longum 105-A to enterocyte-like Caco-2 cells and phagocytosis by macrophages
Source: Gut Pathog. 2017 May 1;9:27. doi: 10.1186/s13099-017-0177-x (PMC5412050; doi:10.1186/s13099-017-0177-x)

## Slide 1
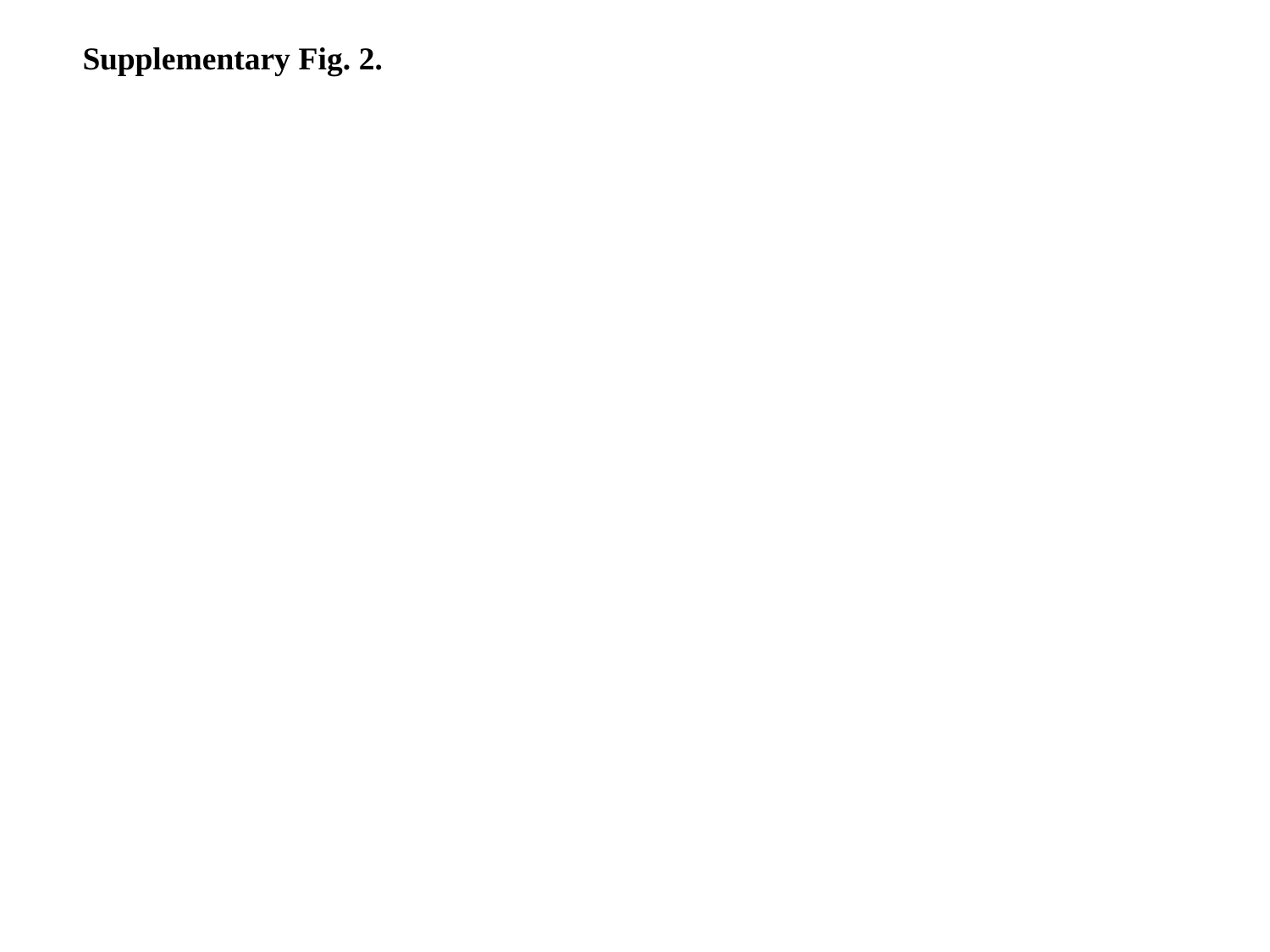

Supplementary Fig. 2.

Supplement: Supplementary file 1 — Additional file 1: Figure S2. SOSUI prediction of the membrane protein in B. longum 105-A. The protein of the gene BL105A_0405 (cpsD) to BL105A_0407 and BL105A_0414 to BL105A_0415 are the membrane protein. [file 13099_2017_177_MOESM1_ESM.pptx]

## Slide 1
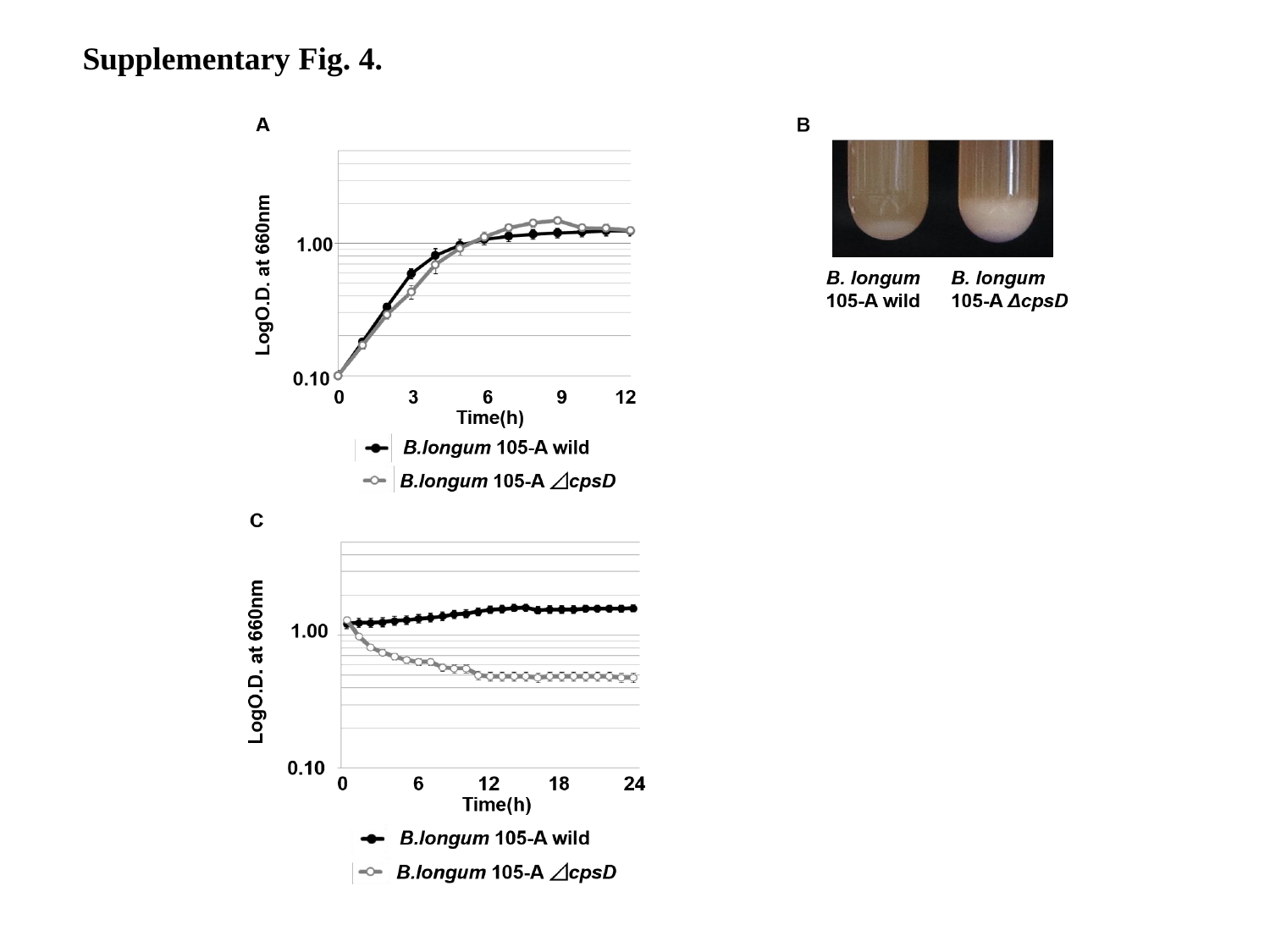

Supplementary Fig. 4.

Supplement: Supplementary file 2 — Additional file 2: Figure S4. Characteristic features of B. longum wild-type strain and its ∆cpsD mutant in growth medium. (a) The growth curves of the wild-type B. longum strain and the B. longum 105-A ∆cpsD mutant strain were similar. (b) The cells of ∆cpsD mutant quickly sediment after stationery phase in liquid medium while the wild-type strain remained in suspension. (c) Measurements OD 660nm of wild-type B. longum strain and the B. longum 105-A ∆cpsD mutant over a panel time point grown ii liquid culture without agitation; the detected decrease in OD values for B. longum 105-A ∆cpsD mutant is due to cell sedimentation. [file 13099_2017_177_MOESM2_ESM.pptx]

## Slide 1
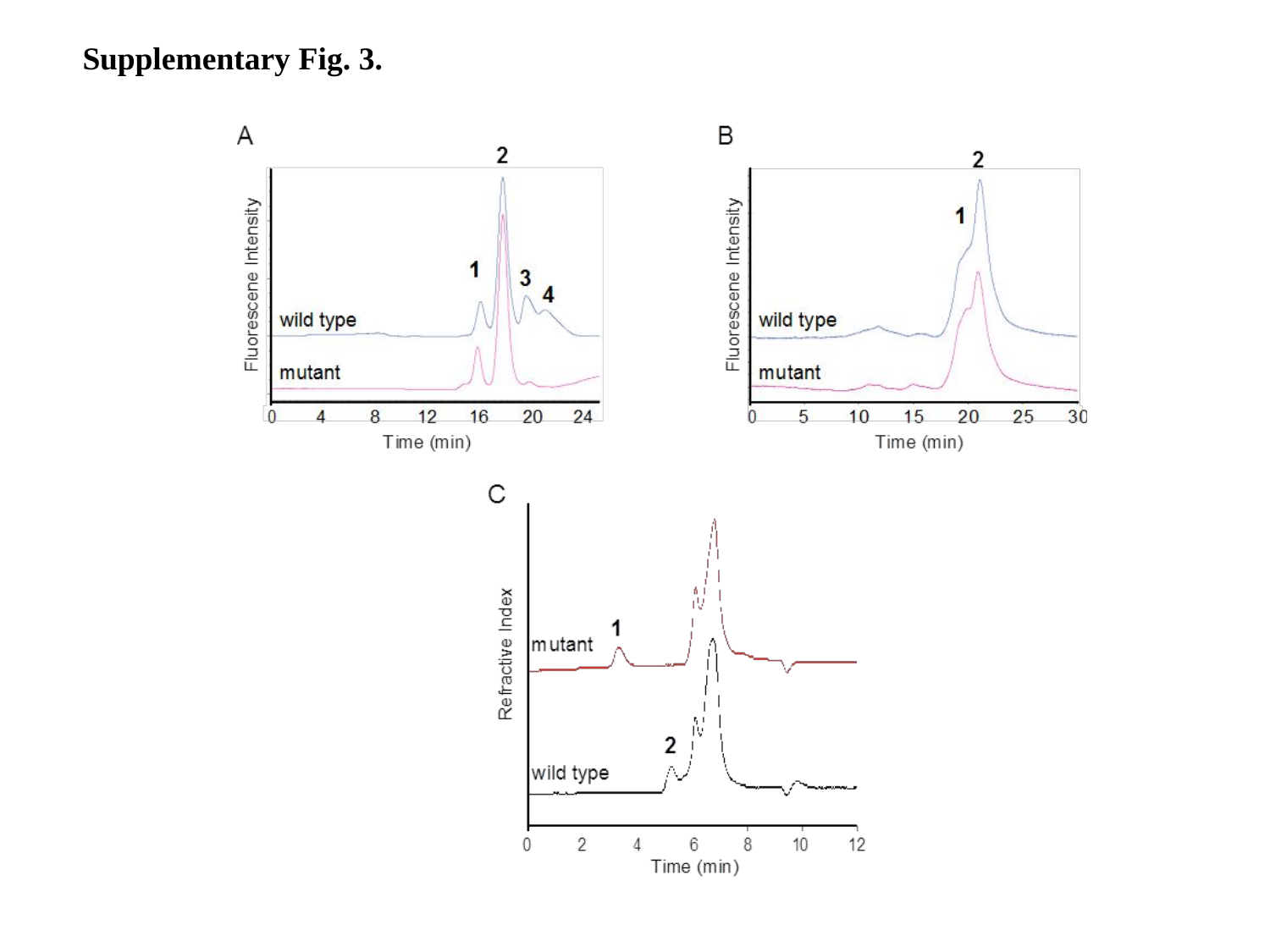

Supplementary Fig. 3.

Supplement: Supplementary file 3 — Additional file 3: Figure S3. HPLC analysis of EPS derived from B. longum 105-A wild-type its ΔcpsD mutant. A: Chromatogram of the monosaccharides in hydrolysates of the EPS from wild-type B. longum 105-A (blue line) and B. longum 105-A ΔcpsD mutant (red line). The chromatography was performed with Asahipak GS-220 HQ column (300 × 7.5 mm) and monitored by a fluorescent detector (Excitation: 331 nm, Emission: 383 nm). Peak 1, galacturonic acid; Peak 2, glucose and galactose; Peak 3 and 4, are unknown. B: Chromatogram of the monosaccharides in hydrolysates of the EPS from wild-type B. longum 105-A (blue line) and B. longum 105-A ΔcpsD mutant (red line) for separating the glucose and galactose. The chromatography was performed with YMC-Pack NH2 column (250 × 4.6 mm) and monitored by a fluorescent detector (Excitation: 331 nm, Emission: 383 nm). The ratio of Glc : Gal was 1 : 1.48 in the wild-type, while it was 1 : 1.76 in its ∆cpsD mutant. Peak 1, glucose; Peak 2, galactose. C: Chromatogram of the molecular weight distribution of the EPS from wild-type B. longum 105-A (black line) and B. longum 105-A ΔcpsD mutant (red line). The chromatography was performed with SUGAR KS-804 column (300 × 8.0 mm) and monitored by a refractive index detector. Average molecular weight: Peak 1, 500 kDa; Peak 2, 200 kDa. [file 13099_2017_177_MOESM3_ESM.pptx]

## Slide 1
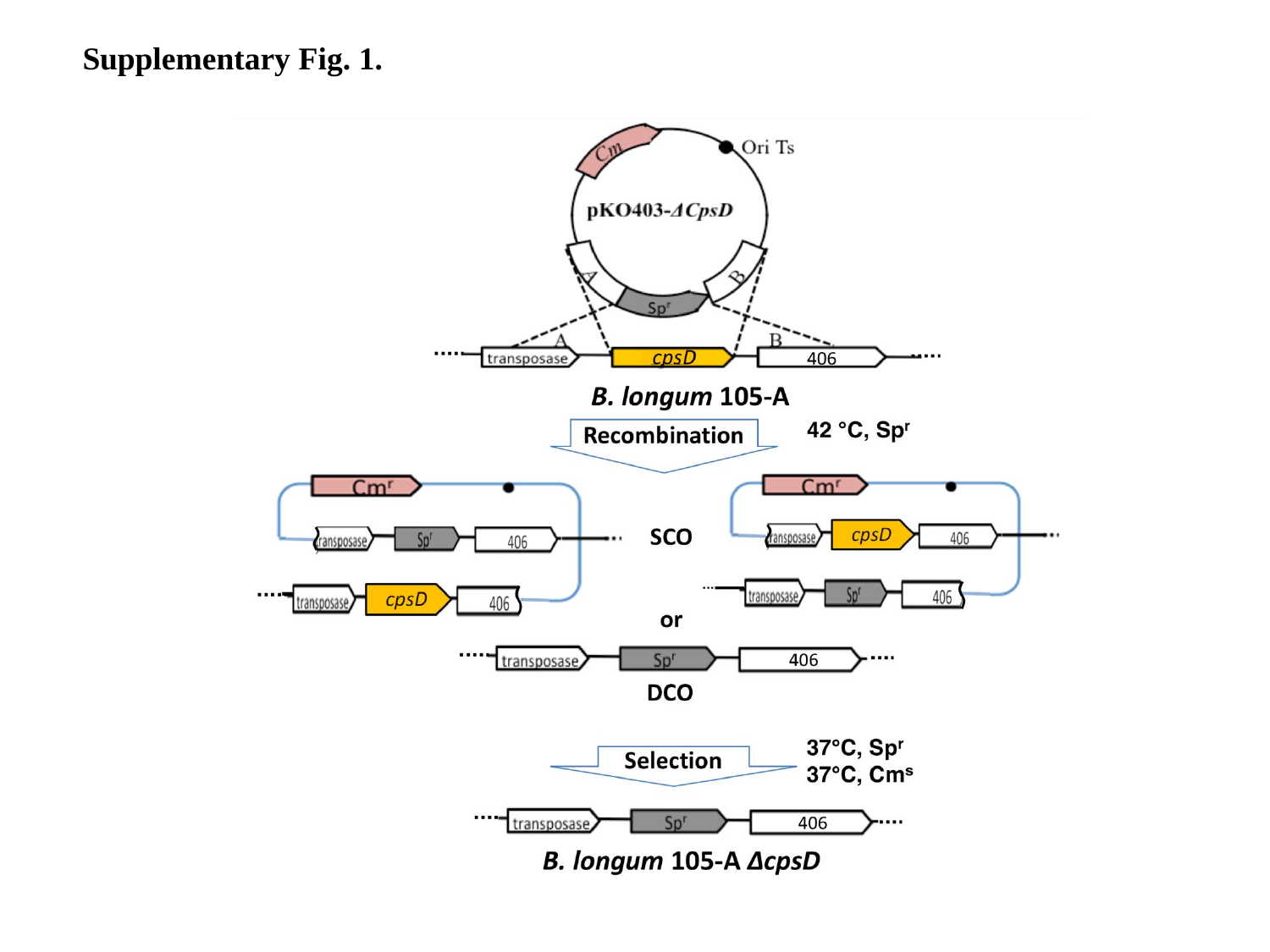

Supplementary Fig. 1.

Supplement: Supplementary file 5 — Additional file 5: Figure S1. Schematic presentation of gene Knockout construction of cpsD. About 1 kb length upstream (BL105A_403, transposase) and downstream (BL105A_406, 406) regions were amplified and introduced into the franking regions of Spr marker on pKO403-Cm (1) (Sakaguchi), which carries temperature sensitive reprocation origin (Ori Ts) and Cmr marker. The obtained plasmid (pKO403-∆cpsD) was introduced into B. longum 105-A, then selected on MRS+Sp plate at 42 °C. Obtained recombinants should consist of Spr and Cmr single cross over (SCO) clones and Spr and Cms double cross over (DCO) clones. DCO clone was selected by by the replica selection with MRS+Sp and Cm plate. Obtained DCO clone was confirmed by PCR and DNA sequencing and designated B. longum 105-A ∆cpsD. [file 13099_2017_177_MOESM5_ESM.pptx]
