# Supplementary material for: Capsular polysaccharide inhibits adhesion of Bifidobacterium longum 105-A to enterocyte-like Caco-2 cells and phagocytosis by macrophages
Source: Gut Pathog. 2017 May 1;9:27. doi: 10.1186/s13099-017-0177-x (PMC5412050; doi:10.1186/s13099-017-0177-x)
Supplement: Supplementary file 4 — Additional file 4: Table S1. Primers used in this study. [file 13099_2017_177_MOESM4_ESM.docx]

# Supplementary Table 1

**Capsular polysaccharide inhibits adhesion of *Bifidobacterium longum* 105-A to enterocyte-like Caco-2 cells and phagocytosis by macrophages**

Amin Tahoun^1, 2＊^, Hisayoshi Masutani^1＊^, Hanem El-Sharkawy^2, 3^, Trudi Gillespie^4^,

Ryo P. Honda^5^, Kazuo Kuwata^6, 7, 8^, Mizuho Inagaki^1, 9^, Tomio Yabe^1, 8, 9^, Izumi Nomura^1^

and Tohru Suzuki^1, 9 #^

**Table S1. Primers used in this study**

| **Primer name** | **Primer set (5’🡪3’)** | **Purpose** |
| --- | --- | --- |
| 105-A *cpsD* Up Fw | ACACAGGGGCGAACTCAAGCAG | Amplify the upstream of *cpsD* |
| 105-A *cpsD* Up Rv | AAGGTGGCTGAGCTGCTTTTGG |  |
| 105-A *cpsD* down Fw | GAATTCCTTCCCGTTTCGCTGTC | Amplify the downstream of *cpsD* |
| 105-A *cpsD* down RV | CTAGCTCGCGGCTTCATCCTG |  |
| 105-A *cpsD* check Fw | GCGACTGTGGGACGAGATC | Confirm the knockout of *cpsD* |
| 105-A *cpsD* check Rv | CCGCTGCAGGAACACTGG |  |
| M13-Fw | GTAAAACGACGGCCAG | Amplify the insert of pUC19 |
| M13-Rv | CAGGAAACAGCTATGAC |  |
| PyrE_term_Rv | ATGGCAGAAACACTCGCACA | Amplify the insert of pKO403 |
| NPE_Fw | TGGAACGTAATAAAAAAAGCGGGC |  |
| BL0005 SP Fw | GAATTCCCATTAAATAATAAAACAA | Confirm the insert of spectinomycine resistance gene |
| BL0005 SP Rv | GGTCGATTTTCGTTCGTGAATACAT |  |
| 105-A *cpsD* rt fw | ATGCCCGATTCATCTCAATCCC | Assess the gene expression of *cpsD* |
| 105-A *cpsD* rt rv | CCTCGTTCTTTTGCATCTCCTCAC |  |
| 105-A 378 rt fw | AAGCCGCATCCAATCACAAGTTCA | Assess the gene expression of BL105A_0406 |
| 105-A 378 rt rv | TTTCCATACCGGCTGCTTGGTC |  |
| 105-A *wzy* rt fw | TGCAATGGTTGGCTATTTGGGTTC | Assess the gene expression of BL105A_0414 |
| 105-A *wzy* rt rv | AAGCCACCTGAAGAATTTGATCGTG |  |
| 105-A *rml* rt fw | AAGTCGGCAACGCTAGGTTCA | Assess the gene expression of BL105A_0424 |
| 105-A *rml* rt rv | TCTTCCCCTCCACCACAAGTTC |  |
| 105-A *rnpA* rt fw | ATCGCCGTTTGGGATTAGCTG | Assess the gene expression of *rnpA* |
| 105-A *rnpA* rt rv | GCCAATACACGAAACCTACGCTTC |  |
| 105-A 380 rt fw | CTGAATGCGCTGATGTGCT | Assess the gene expression of BL105A_0408 |
| 105-A 380 rt rv | TGTCGGTCAAATCGTTCACATCG |  |

**References**

1. Sakaguchi, K., He, J., Tani, S., Kano, Y., and Suzuki, T. (2012) A targeted gene knockout method using a newly constructed temperature-sensitive plasmid mediated homologous recombination in *Bifidobacterium longum*. *Appl. Microbiol. Biotechnol.* **95**, 499–509
